# Strong genetic effect on gout revealed by genetic risk score from meta-analysis of two genome-wide association studies

**DOI:** 10.1007/s13577-024-01138-y

**Published:** 2024-11-11

**Authors:** Akiyoshi Nakayama, Yusuke Kawamura, Masahiro Nakatochi, Yu Toyoda, Mayuko Nakajima, Kazuki Maehara, Mana Kirihara, Seiko Shimizu, Keitaro Matsuo, Hirotaka Matsuo, the Japan Gout Genomics Consortium (Japan Gout), Toru Shimizu, Hiroshi Ooyama, Keiko Ooyama, Mitsuo Nagase, Yuji Hidaka, Ken Yamamoto, Tappei Takada, Kimiyoshi Ichida, Kenji Wakai, Nariyoshi Shinomiya, Miki Ueno, Kimiko Hayano, Hiroshi Nakashima, Mitsunobu Tanaka, Noriyuki Yoshioka, Satoko Iwasawa, Masashi Tsunoda, Yuzo Takada, Takahiro Nakamura, Kyoko Morichika, Miho Miyazawa, Yuka Aoki, Yuka Aoyagi, Mio Horie, Risa Tanaka, Yurino Mori, Shin Fujiwara, Masumi Someya

**Affiliations:** 1 Department of Integrative Physiology and Bio-Nano Medicine, National Defense Medical College, Tokorozawa, Japan; 2 Public Health Informatics Unit, Department of Integrated Health Sciences, Nagoya University Graduate School of Medicine, Nagoya, Japan; 3 Department of Pharmacy, The University of Tokyo Hospital, Tokyo, Japan; 4 Division of Cancer Epidemiology and Prevention, Aichi Cancer Center Research Institute, Nagoya, Japan; 5 Department of Biomedical Information Management, National Defense Medical College Research Institute, National Defense Medical College, Tokorozawa, Japan

**Keywords:** Gout, Hyperuricemia, Genome-wide association study, Genetic risk score, Urate/uric acid, Dysuricemia

## To the Editor

Gout is a common form of arthritis caused by prolonged hyperuricemia or elevated serum uric acid (SUA) levels. Recent studies have revealed that gout/hyperuricemia has much stronger genetic factors for their susceptibility than are seen in many common diseases [1]. With 121,745 Japanese, we previously performed a genome-wide association study (GWAS) on SUA variation [2] that revealed 36 statistically significant (*p* < 5 × 10^–8^) loci. Of these, eight loci (*ABCG2*, *CUX2*, *SLC2A9*, *FAM35A*, *GCKR*, *NRXN2*, *SLC17A1*, and *BCAS3*) were also included in the ten loci reported in another GWAS with clinically defined gout cases [1], but the remaining 28 loci were not.

We therefore examined their associations with gout through a genome-wide meta-analysis that was based on two genotyping data sets which included the Japonica Array and Illumina Array platforms and was conducted with Japanese male subjects, comprising 3,053 clinically defined gout cases and 4,554 normouricemic (SUA ≤ 7.0 mg/dl) controls as previously described [1]. The significance level α was set to a *p* value of < 1.79 × 10^–3^ (= 0.05/28 with Bonferroni correction).

We identified nine significant gout loci among 28 loci (Table 1, Supplementary Table S1). Five loci (*UNCX-MICALL2*, *BICC1*, *EMX2-RAB11FIP2*, *IGF1R*, and *NFAT5*) were identified for the first time in clinically defined gout cases. Remaining four loci (*PDZK1*, *LRP2*, *PRDM8-FGF5*, and *MLXIPL* (*BAZ1B*)) were previously evidenced as gout loci by the association analyses with clinically defined gout cases [3–6]. Taking into account the strong genetic contributor to gout, we then computed genetic risk scores (GRSs) in order to estimate the predictive ability using only the genetic variants of the 19 loci, that is, the present nine loci and the previous ten gout loci [1] (see Supplementary Methods and Table S2). The areas under the receiver-operating characteristic curve (AUC) were respectively 0.765 and 0.749 (Supplementary Fig. S1) from the genotyping data of the Japonica Array and Illumina Array platforms.

**Table 1:** Gout loci identified in the present genome-wide meta-analyses

SNP^a^	Locus	Chr	Position^b^	Gene^c^	Risk allele	Non-risk allele	OR (95%CI)	*p* value	*I*^*2*^ (%)	HetP
rs1797052	1q21.1	1	145,727,683	*PDZK1*	T	C	1.24 (1.14, 1.35)	5.99 × 10^–7^	61.9	0.105
rs16856823	2q31.1	2	170,200,452	*LRP2*	T	A	1.16 (1.06, 1.27)	9.81 × 10^–4^	0	0.456
rs10857147	4q21.21	4	81,181,072	*PRDM8-FGF5*	A	T	1.20 (1.12, 1.30)	1.69 × 10^–6^	0	0.724
rs17145750	7q11.23	7	73,026,378	*MLXIPL/CHREBP* (*BAZ1B*)	C	T	1.30 (1.16, 1.47)	1.26 × 10^–5^	0	0.634
**rs13230625**	**7p22.3**	**7**	**1,286,244**	***UNCX-MICALL2***	**A**	**G**	**1.14 (1.06, 1.22)**	**6.04 × 10**^**–4**^	**0**	**0.428**
**rs9416703**	**10q21.1**	**10**	**60,283,008**	***BICC1***	**C**	**A**	**1.14 (1.07, 1.22)**	**1.40 × 10**^**–4**^	**88**	**0.004**
**rs1886603**	**10q26.11**	**10**	**119,482,303**	***EMX2-RAB11FIP2***	**A**	**G**	**1.12 (1.04, 1.20)**	**1.70 × 10**^**–3**^	**0**	**0.417**
**rs4966024**	**15q26.3**	**15**	**99,295,570**	***IGF1R***	**G**	**A**	**1.17 (1.09, 1.25)**	**1.04 × 10**^**–5**^	**0**	**0.539**
**rs244423**	**16q22.1**	**16**	**69,610,002**	***NFAT5***	**A**	**G**	**1.24 (1.13, 1.37)**	**1.24 × 10**^**–5**^	**0**	**0.354**

^a^dbSNP rs number

^b^SNP positions are based on NCBI human genome reference sequence Build hg19

^c^Loci identified for the first time in clinically defined gout cases are shown in **bold**

The significance level α was set to a *p* value of < 1.79 × 10^–3^ (= 0.05/28 with Bonferroni correction)

*SNP* Single nucleotide polymorphism, *Chr* Chromosome, *OR* Odds ratio, *95%CI* 95% Confidence interval, *HetP* p value for heterogeneity

**Fig. 1:**
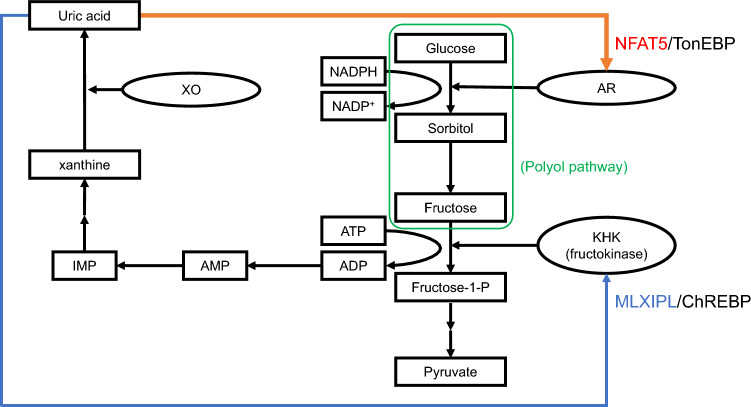
Involvement of NFAT5 in uric acid production. Fructose is generated from glucose via aldose reductase (AR) through polyol pathway. Fructose is then converted to fructose-1-phosphate (Fructose-1-P) via ketohexokinase (KHK, also known as fructokinase) with consuming adenosine triphosphate (ATP). Dephosphorylated ATP, or adenosine diphosphate (ADP) and adenosine monophosphate (AMP), is metabolized to inosine monophosphate (IMP), and finally to uric acid via xanthine oxidase (XO). Uric acid in turn stimulates transcription factors, NFAT5/TonEBP and MLXIPL/ChREBP, which respectively enhance AR and KHK and increase uric acid level in a positive feedback loop. See the Supplementary Discussion for further details

The present study revealed nine significantly associated loci with gout with odds ratios of 1.12–1.30 (Table 1). In spite of searching for the functions of the proteins encoded by the five loci identified in the present study (see Supplementary Discussion), their physiological and pathophysiological functions on urate handling and/or the susceptibility of gout remained unclear, except for* IGF1R* [7] and *NFAT5* (Fig. 1). This is the first report that indicates the association between gout and *NFAT5*, which deserves attention because NFAT5/TonEBP stimulates uric acid production via aldose reductase (AR) [8]: glucose generates fructose via AR and fructose produces uric acid, and uric acid in turn stimulates NFAT5 which enhance AR in a positive feedback loop (Fig. 1, Supplementary Discussion). In addition to the higher heterogeneity of *BICC1* than that of other loci, further analyses with larger populations are necessary to elucidate its association and their precise involvement to be able to develop novel therapies to prevent gout attacks and/or lower SUA levels. Nevertheless, the present study also revealed high values of the AUC for GRS, using the 19 loci alone, which reached approximately 0.75. This value is higher than the AUC of 0.67 from a previous report on gout based on 114 SUA-associated SNPs identified in European ancestry [9]. This discrepancy can be partly explained by the fact that the Japanese population has been subjected to significant selection pressure for gout susceptibility [1] although further evaluations are necessary for the present study using independent populations. Numerous loci and/or evaluations employing conventional risk factors are usually required to reach such a high AUC value in other common diseases. An AUC of ≥ 0.75 suggests that the test is useful for screening to identify individuals who are at increased risk of disease [10]. In light of these points, the evaluation of gout susceptibility using these 19 loci alone could be a unique yet feasible method of applying genome-personalized medicine/prevention or precision medicine.

## Supplementary Information

Below is the link to the electronic supplementary material.

Supplementary file1 (DOCX 179 KB)

Supplementary file2 (XLSX 23 KB)

## Data Availability

Data are available upon reasonable request to the corresponding author.
