# Supplementary material for: Strong genetic effect on gout revealed by genetic risk score from meta-analysis of two genome-wide association studies
Source: Hum Cell. 2024 Nov 11;38(1):16. doi: 10.1007/s13577-024-01138-y (PMC11554751; doi:10.1007/s13577-024-01138-y)
Supplement: Supplementary file 1 — Supplementary file1 (DOCX 179 KB) [file 13577_2024_1138_MOESM1_ESM.docx]

Supplementary Information

**Title:**

Strong genetic effect on gout revealed by genetic risk score from meta-analysis of two genome-wide association studies

**Journal Name:**

*Human Cell*

**Authors:**

Akiyoshi Nakayama^1^, Yusuke Kawamura^1^, Masahiro Nakatochi^2^, Yu Toyoda^1,3^, Mayuko Nakajima^1^, Kazuki Maehara^1^, Mana Kirihara^1^, Seiko Shimizu^1^, Keitaro Matsuo^4^, Hirotaka Matsuo^1,5,*^ on behalf of the Japan Gout Genomics Consortium (Japan Gout)

**Affiliations:**

1. Department of Integrative Physiology and Bio-Nano Medicine, National Defense Medical College, Tokorozawa, Japan
2. Public Health Informatics Unit, Department of Integrated Health Sciences, Nagoya University Graduate School of Medicine, Nagoya, Japan
3. Department of Pharmacy, The University of Tokyo Hospital, Tokyo, Japan
4. Division of Cancer Epidemiology and Prevention, Aichi Cancer Center Research Institute, Nagoya, Japan
5. Department of Biomedical Information Management, National Defense Medical College Research Institute, National Defense Medical College, Tokorozawa, Japan

***Correspondence to:**

H. Matsuo (matsuo29@gmail.com).

Contents:

Supplementary Table S1: All 28 loci that are associated with serum uric acid level (SUA) examined in the present genome-wide meta-analyses for gout. [see *Supplementary Excel File*]

Supplementary Table S2: 19 SNPs used to calculate genetic risk score

Supplementary Figure S1: Estimation of predictive ability based on the genetic risk score (GRS) of 19 gout-associated variants

Supplementary Method

Supplementary Discussion

Supplementary References

**Supplementary Table S1** All 28 loci that are associated with serum uric acid level (SUA) examined in the present genome-wide meta-analyses for gout

[see *Supplementary Excel File*]

**Supplementary Table** **S2** 19 SNPs used for the calculation of genetic risk score

| SNP^a^ | Locus | Chr | Position^b^ | Gene | Risk Allele | Non-risk Allele | *w_i_* | Reference |
| --- | --- | --- | --- | --- | --- | --- | --- | --- |
| rs1797052 | 1q21.1 | 1 | 145727683 | *PDZK1* | T | C | 0.2164 | Present study |
| rs16856823 | 2q31.1 | 2 | 170200452 | *LRP2* | T | A | 0.1491 | Present study |
| rs10857147 | 4q21.21 | 4 | 81181072 | *PRDM8-FGF5* | A | T | 0.1856 | Present study |
| rs13230625 | 7q11.23 | 7 | 1286244 | *MLXIPL* (*BAZ1B*) | A | G | 0.1285 | Present study |
| rs17145750 | 7p22.3 | 7 | 73026378 | *UNCX-MICALL2* | C | T | 0.264 | Present study |
| rs9416703 | 10q21.1 | 10 | 60283008 | *BICC1* | C | A | 0.1305 | Present study |
| rs1886603 | 10q26.11 | 10 | 119482303 | *EMX2-RAB11FIP2* | A | G | 0.1112 | Present study |
| rs4966024 | 15q26.3 | 15 | 99295570 | *IGF1R* | G | A | 0.1551 | Present study |
| rs244423 | 16q22.1 | 16 | 69610002 | *NFAT5* | A | G | 0.2156 | Present study |
| rs3129500 | 10q23.2 | 10 | 88915107 | *SHLD2* (*FAM35A*) | G | A | 0.3183 | Reference [1] |
| rs145954970 | 11q13.1 | 10 | 64273830 | *SLC22A11* | C | G | 2.5203 | Reference [1] |
| rs671 | 12q24.12 | 10 | 112241766 | *ALDH2* | G | A | 0.658 | Reference [1] |
| rs76499759 | 13q22.1 | 11 | 73568511 | *PIBF1* | A | G | 0.2377 | Reference [1] |
| rs9926388 | 16p12.3 | 12 | 20558441 | *ACSM2B* | A | G | 0.2136 | Reference [1] |
| rs1010269 | 17q23.2 | 13 | 59448945 | *BCAS3* | G | A | 0.2157 | Reference [1] |
| rs1260326 | 2p23.3 | 15 | 27730940 | *GCKR* | T | C | 0.2589 | Reference [1] |
| rs3775946 | 4p16.1 | 16 | 9995256 | *SLC2A9* | G | A | 0.4901 | Reference [1] |
| rs4148155 | 4q22.1 | 16 | 89054667 | *ABCG2* | G | A | 0.804 | Reference [1] |
| rs2817188 | 6p22.2 | 17 | 25807603 | *SLC17A1* | G | A | 0.3013 | Reference [1] |

^a^ dbSNP rs number.

^b^ SNP positions are based on NCBI human genome reference sequence Build hg19.

Abbreviations: *SNP*: single nucleotide polymorphism, *Chr*: chromosome, *w_i_*: weight.


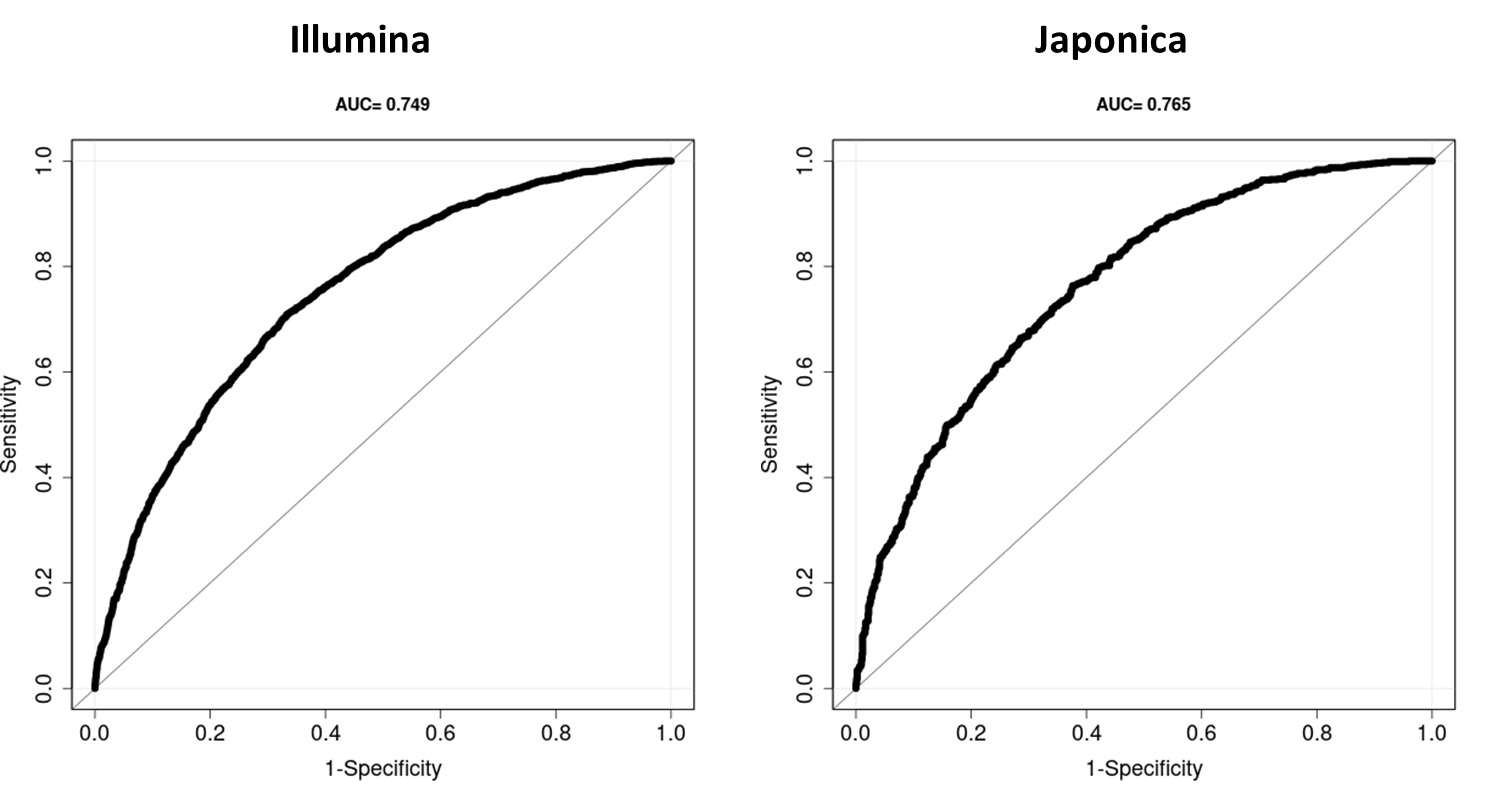


**Supplementary Figure S1** Estimation of predictive ability based on the genetic risk score (GRS) of 19 gout-associated variants.

The area under the receiver-operating characteristic curve (AUC) of GRS by the 19 loci alone reached approximately 0.75 in both genotyping arrays, indicating genotyping of only 19 SNPs to be useful for screening to identify individuals at increased gout risk.

**Supplementary method**

**Genetic risk score (GRS)**

The GRS of 19 gout-associated SNPs (Supplementary Table S3) were constructed. The value of GRS*_i_* for individual *i* was calculated by applying the following equation,

$$\mathrm{GRS}_{i}=\sum_{j}^{m} w_{j}G_{ij}$$

where *w_j_* is the effect size (ln odds ratio) of SNP _j_ and *G_ij_* is the number of risk alleles of the genotype for SNP *j* in individual *i*. The effect sizes were obtained from the above meta-analysis. The GRS*_i_* was calculated using the above formula for each of the 3,104 total cases (1,048 for the Japonica Array and 2,056 for the Illumina Array) and 6,081 controls (1,179 for the Japonica Array and 4,902 for the Illumina Array) for which the meta-analysis was performed. A receiver-operating characteristic curve (ROC) analysis was performed to assess the predictive ability of the GRS in each of the data sets obtained from the Illumina and Japonica arrays. The area under the curve (AUC) was calculated using the ROC analysis. In this section, all analyses were performed by R version 3.6.0 and the pROC package.

**Supplementary Discussion: function of the proteins encoded by the nine loci identified in the present study**

***UNCX* and *MICALL2***

*UNCX* (*UNC homeobox*) encodes a paired-type homeobox transcription factor and plays a role in kidney development as well as in differentiation of the nervous system [1]. A meta-analysis [2] of GWAS from 71,149 East Asian individuals revealed that rs10277115 of *UNCX* had an association with kidney function-related traits including blood urea nitrogen, serum creatinine, estimated glomerular filtration rate with creatinine (eGFRcreat), and chronic kidney disease (CKD) with genome-wide significance, and with uric acid being of nominal significance.

*MICALL2* (*MICAL like 2*) encodes a protein which interacts with both Rab8 and Rab13 [3]. When the Rab8-MICALL2 complex resides at the perinuclear recycling/storage compartments, it mediates the recycling of E-cadherin to the plasma membrane and the assembly of adherence junctions; it also regulates the recycling of claudins and occludin to the plasma membrane and the formation of tight junctions at the plasma membrane [3]. A recent study has revealed MICALL2 to be a novel prognostic biomarker that is correlated with inflammation and T cell exhaustion of renal clear cell carcinoma [4].

Taking into account that two-thirds of serum uric acid is excreted from the kidney and the remainder from the intestine, and that gout flare is accompanied by severe inflammation, it is possible that either or both of these proteins (UNCX and MICALL2) have an association with gout [5].

***BICC1***

*BICC1* (*BicC family RNA binding protein 1*) encodes an RNA-binding protein which recruits a deadenylase to specific mRNAs, localizes mRNA, and leads to translational repression in *Drosophila* [6]. Bicc1 is expressed in mouse inner medullary collecting duct (IMCD) cells [7] and its expression is stimulated by PKD1 (polycystic kidney disease 1), mutations of which are known to cause autosomal dominant polycystic kidney diseases (ADPKD) [8]. Two children with a renal disease characterized by renal cysts and dysplasia were reported to have two heterozygous *BICC1* mutations that affect functional domains of BICC1 [9]. In addition to urate levels, a previous GWAS [10] with 229,086 European and 178,726 Japanese noted that *BICC1* also has association with the use of diuretics. BICC1 therefore plays roles in kidney development and function, which would include urate handling and ultimately gout.

***EMX2* and *RAB11FIP2***

The *EMX2* (*empty spiracles homeobox 2*) gene, similar to *UNCX*, encodes a homeobox-containing transcription factor. The kidneys, ureters, gonads and genital tracts were completely missing in *Emx2* mutant mice [11]. EMX2 appears to suppress carcinogenesis and/or tumor growth in several tumors [12-14], and may be a therapeutic target for their treatment.

*RAB11FIP2* (*RAB11 family interacting protein 2*) is involved in endocytic vesicular transport by making a triple complex among myosin Vb, Rab11a, and RAB11FIP2, and in the recycling of many proteins, including the NPC1L1/SLC65A2 transporter that absorbs cholesterol in the intestine [15]. Rab11FIP2 is also reported to promote the metastasis of gastric cancer cells [16], and its knockdown cell lines inhibit migration and invasion of nasopharyngeal carcinoma via suppression of Rho GTPase signaling [17]. While its associations with urate transporters are not known, Rab11FIP2 might be involved in their recycling, which affects urate handling.

***IGF1R***

The *IGF1R* (*insulin like growth factor 1 receptor*) gene encodes a receptor which binds and activated by insulin-like growth factor 1, and IGF1R then transduces an anti-apoptotic signal enhancing cell survival and plays a central role in cell cycle progression and transformation [18]. Beta cell-specific *Igf1r* knock-out in mice displayed defective glucose stimulated insulin secretion and impaired glucose tolerance [18]. Insulin and hyperinsulinemia are well-known to reduce renal fractional excretion of uric acid (FE_UA_) and play a key role in the genesis of hyperuricemia and gout [19]. Phipps-Green *et al.* reported the association with gout and *IGF1R* for the first time [20]. *IGF1R* therefore should have relationship with SUA and gout.

***NFAT5***

*NFAT5* (*nuclear factor of activated T cells 5*) also encodes a transcription factor. NFAT5/TonEBP (Fig. 1) is known to regulate aldose reductase (AR) expression and synthesis [21, 22]. Previous studies [23-26] have reported that 1) AR converts glucose to sorbitol, which is followed by the conversion of sorbitol to fructose by sorbitol dehydrogenase (SDH). Endogenous fructose can be produced only via this polyol (AR-SDH) pathway in humans. 2) Although dietary fructose constitutes a major source of fructose in humans, endogenous fructose has a pathogenic role in the fatty liver induced by high-glycemic and high-salt diets. 3) Uric acid is generated from ATP consumption by ketohexokinase (KHK, also known as fructokinase) which metabolizes both endogenous and exogenous fructose, and also acts as an amplifying pathway to stimulate upstream enzymes including AR by activating NFAT5. 4) Uric acid also stimulates KHK by activation of another transcription factor, MLXIPL/ChREBP, to generate more fructose. In summary, fructose is generated from glucose via aldose reductase (AR). Uric acid is produced from fructose via ketohexokinase. Uric acid in turn stimulates NFAT5/TonEBP and MLXIPL/ChREBP, which respectively enhance AR and KHK to increase uric acid as positive feedback (Fig. 1). A recent meta-analysis [27] also showed a gout-associated intergenic SNP (rs62052820) in *CYB5B-MIR1538* locus, which is close to the intronic SNP (rs244423) in *NFAT5* identified in the present study. In addition to our previous report on the association between gout and *MLXIPL* (*BAZ1B*) [28, 29], the present study therefore reveals the genetic involvement of *NFAT5* in gout.

Thus, the present study is the first report that indicates the association between gout and *NFAT5*, which deserves attention because NFAT5 stimulates uric acid production in a positive feedback loop.

**Supplementary references**

1. Mansouri A, Yokota Y, Wehr R, Copeland NG, Jenkins NA, Gruss P. Paired-related murine homeobox gene expressed in the developing sclerotome, kidney, and nervous system. Dev Dyn. 1997;210(1):53-65. doi:10.1002/(SICI)1097-0177(199709)210:1<53::AID-AJA6>3.0.CO;2-0.

2. Okada Y, Sim X, Go MJ, Wu JY, Gu D, Takeuchi F et al. Meta-analysis identifies multiple loci associated with kidney function-related traits in east Asian populations. Nat Genet. 2012;44(8):904-9. doi:10.1038/ng.2352.

3. Nishimura N, Sasaki T. Rab family small G proteins in regulation of epithelial apical junctions. Front Biosci (Landmark Ed). 2009;14:2115-29. doi:10.2741/3366.

4. Lin W, Chen W, Zhong J, Ueki H, Xu A, Watanabe M et al. Identification of MICALL2 as a Novel Prognostic Biomarker Correlating with Inflammation and T Cell Exhaustion of Kidney Renal Clear Cell Carcinoma. J Cancer. 2022;13(4):1214-28. doi:10.7150/jca.66922.

5. Ichida K, Matsuo H, Takada T, Nakayama A, Murakami K, Shimizu T et al. Decreased extra-renal urate excretion is a common cause of hyperuricemia. Nat Commun. 2012;3:764. doi:10.1038/ncomms1756.

6. Snee MJ, Macdonald PM. Bicaudal C and trailer hitch have similar roles in gurken mRNA localization and cytoskeletal organization. Dev Biol. 2009;328(2):434-44. doi:10.1016/j.ydbio.2009.02.003.

7. Stagner EE, Bouvrette DJ, Cheng J, Bryda EC. The polycystic kidney disease-related proteins Bicc1 and SamCystin interact. Biochem Biophys Res Commun. 2009;383(1):16-21. doi:10.1016/j.bbrc.2009.03.113.

8. Rothé B, Gagnieux C, Leal-Esteban LC, Constam DB. Role of the RNA-binding protein Bicaudal-C1 and interacting factors in cystic kidney diseases. Cell Signal. 2020;68:109499. doi:10.1016/j.cellsig.2019.109499.

9. Kraus MR, Clauin S, Pfister Y, Di Maio M, Ulinski T, Constam D et al. Two mutations in human BICC1 resulting in Wnt pathway hyperactivity associated with cystic renal dysplasia. Hum Mutat. 2012;33(1):86-90. doi:10.1002/humu.21610.

10. Sakaue S, Kanai M, Tanigawa Y, Karjalainen J, Kurki M, Koshiba S et al. A cross-population atlas of genetic associations for 220 human phenotypes. Nat Genet. 2021;53(10):1415-24. doi:10.1038/s41588-021-00931-x.

11. Miyamoto N, Yoshida M, Kuratani S, Matsuo I, Aizawa S. Defects of urogenital development in mice lacking Emx2. Development. 1997;124(9):1653-64. doi:10.1242/dev.124.9.1653.

12. Monnier A, Boniface R, Bouvet R, Etcheverry A, Aubry M, Avril T et al. The expression of EMX2 lead to cell cycle arrest in glioblastoma cell line. BMC Cancer. 2018;18(1):1213. doi:10.1186/s12885-018-5094-y.

13. Falcone C, Daga A, Leanza G, Mallamaci A. Emx2 as a novel tool to suppress glioblastoma. Oncotarget. 2016;7(27):41005-16. doi:10.18632/oncotarget.9322.

14. Zhang Y, Cao G, Yuan QG, Li JH, Yang WB. Empty Spiracles Homeobox 2 (EMX2) Inhibits the Invasion and Tumorigenesis in Colorectal Cancer Cells. Oncol Res. 2017;25(4):537-44. doi:10.3727/096504016x14756640150695.

15. Chu BB, Ge L, Xie C, Zhao Y, Miao HH, Wang J et al. Requirement of myosin Vb.Rab11a.Rab11-FIP2 complex in cholesterol-regulated translocation of NPC1L1 to the cell surface. J Biol Chem. 2009;284(33):22481-90. doi:10.1074/jbc.M109.034355.

16. Dong W, Qin G, Shen R. Rab11-FIP2 promotes the metastasis of gastric cancer cells. Int J Cancer. 2016;138(7):1680-8. doi:10.1002/ijc.29899.

17. Feng G, Qin L, Liao Z, Xiao X, Li B, Cui W et al. Knockdown Rab11-FIP2 inhibits migration and invasion of nasopharyngeal carcinoma via suppressing Rho GTPase signaling. J Cell Biochem. 2020;121(2):1072-86. doi:10.1002/jcb.29344.

18. Kottgen A, Albrecht E, Teumer A, Vitart V, Krumsiek J, Hundertmark C et al. Genome-wide association analyses identify 18 new loci associated with serum urate concentrations. Nat Genet. 2013;45(2):145-54. doi:10.1038/ng.2500.

19. Mandal AK, Leask MP, Estiverne C, Choi HK, Merriman TR, Mount DB. Genetic and Physiological Effects of Insulin on Human Urate Homeostasis. Front Physiol. 2021;12(1153):713710. doi:10.3389/fphys.2021.713710.

20. Phipps-Green AJ, Merriman ME, Topless R, Altaf S, Montgomery GW, Franklin C et al. Twenty-eight loci that influence serum urate levels: analysis of association with gout. Ann Rheum Dis. 2016;75(1):124-30. doi:10.1136/annrheumdis-2014-205877.

21. Na KY, Woo SK, Lee SD, Kwon HM. Silencing of TonEBP/NFAT5 transcriptional activator by RNA interference. J Am Soc Nephrol. 2003;14(2):283-8. doi:10.1097/01.asn.0000045050.19544.b2.

22. Woo SK, Lee SD, Kwon HM. TonEBP transcriptional activator in the cellular response to increased osmolality. Pflugers Arch. 2002;444(5):579-85. doi:10.1007/s00424-002-0849-2.

23. Lanaspa MA, Sanchez-Lozada LG, Cicerchi C, Li N, Roncal-Jimenez CA, Ishimoto T et al. Uric acid stimulates fructokinase and accelerates fructose metabolism in the development of fatty liver. PLoS ONE. 2012;7(10):e47948. doi:10.1371/journal.pone.0047948.

24. Lanaspa MA, Ishimoto T, Li N, Cicerchi C, Orlicky DJ, Ruzycki P et al. Endogenous fructose production and metabolism in the liver contributes to the development of metabolic syndrome. Nat Commun. 2013;4:2434. doi:10.1038/ncomms3434.

25. Lanaspa MA, Kuwabara M, Andres-Hernando A, Li N, Cicerchi C, Jensen T et al. High salt intake causes leptin resistance and obesity in mice by stimulating endogenous fructose production and metabolism. Proc Natl Acad Sci U S A. 2018;115(12):3138-43. doi:10.1073/pnas.1713837115.

26. Sanchez-Lozada LG, Andres-Hernando A, Garcia-Arroyo FE, Cicerchi C, Li N, Kuwabara M et al. Uric acid activates aldose reductase and the polyol pathway for endogenous fructose and fat production causing development of fatty liver in rats. J Biol Chem. 2019;294(11):4272-81. doi:10.1074/jbc.RA118.006158.

27. Zhou W, Kanai M, Wu KH, Rasheed H, Tsuo K, Hirbo JB et al. Global Biobank Meta-analysis Initiative: Powering genetic discovery across human disease. Cell Genom. 2022;2(10):100192. doi:10.1016/j.xgen.2022.100192.

28. Chang SJ, Toyoda Y, Kawamura Y, Nakamura T, Nakatochi M, Nakayama A et al. A meta-analysis of genome-wide association studies using Japanese and Taiwanese has revealed novel loci associated with gout susceptibility. Hum Cell. 2022;35(2):767-70. doi:10.1007/s13577-021-00665-2.

29. Kawaguchi M, Nakayama A, Aoyagi Y, Nakamura T, Shimizu S, Kawamura Y et al. Both variants of A1CF and BAZ1B genes are associated with gout susceptibility: a replication study and meta-analysis in a Japanese population. Hum Cell. 2021;34(2):293-9. doi:10.1007/s13577-021-00485-4.
